# Suppression of immune regulatory cells with combined therapy of celecoxib and sunitinib in renal cell carcinoma

**DOI:** 10.18632/oncotarget.13774

**Published:** 2016-12-02

**Authors:** Qi Zhao, Jianming Guo, Guomin Wang, Yiwei Chu, Xiaoyi Hu

**Affiliations:** ^1^ Department of Urology, Zhongshan Hospital, Fudan University, Shanghai, 200032, China; ^2^ Department of Immunology, Shanghai Medical College of Fudan University, Shanghai, 200032, China

**Keywords:** tyrosine kinase inhibitor, renal cell carcinoma, cyclooxygenase-2 inhibitor, myeloid-derived suppressor cell, regulatory T cell

## Abstract

**Objective:**

To observe the the potential benefit of sunitinib in combination with cyclooxygenase-2(COX-2) inhibitor in renal cell carcinoma therapy.

**Methods:**

769-p cell lines were treated with sunitinib, celecoxib, or in combination at different concentrations respectively. We investigated the expression of granulocyte-macrophage colony stimulating factor (GM-CSF) in 769-p and cell proliferation *in vitro*. BALB/c mice implanted with Renca cells were divided into 4 groups and administered orally by gavage with sunitinib, COX-2 inhibitor (celecoxib) monotherapy or combination, and PBS respectively. Tumor growth and animal survival were observed. The myeloid-derived suppressor cells (MDSCs) and regulatory T cells (Tregs) in peripheral blood and spleen were determined by flow cytometry. The MDSCs protein was extracted for STAT3 analysis by western blot.

**Results:**

769-p cell lines were suppressed in a dose and time-dependent manner. The expression of GM-CSF was substantially inhibited by celecoxib and sunitinib. Combination of sunitinib and celecoxib *in vivo* could effectively reduce the MDSCs than those in control group. Meanwhile, the CD4^+^ lymphocytes were strongly increased and the expression of signal transducer and activator of transcription 3 (STAT3) in MDSCs were significantly reduced.

**Conclusion:**

Combination therapy with sunitinib and celecoxib intensified the curative effects to renal cell carcinoma by suppressing immune regulatory cells.

## INTRODUCTION

In recent years, the application of targeted therapy of VEGF receptor tyrosine kinase inhibitors (VEGFR-TKI) are gradually replacing the conventional immunotherapy and become a standard option in the treatment of metastatic renal cell carcinoma (mRCC). However, the complete responses can hardly achieved with this approach and many patients develop drug resistance eventually. Several investigations showed that a novel anti-inflammation medicine, COX-2 inhibitor, disclosed potent anti-tumor activity in certain murine tumor models by extending the effectiveness of VEGFR inhibition in human renal cell carcinoma xenografts [1]. Despite comprehensive studies being conducting, the mechanisms of combined treatment of COX-2 inhibition with VEGF-targeted drugs are not fully understood.

The process of tumor angiogenesis may be affected by the inhibition of VEGF/VEGFR signaling pathway, as well as the stimulation of anti-tumor immune responses [2, 3]. Angiogenesis and immunosuppression are closely linked in the tumor microenvironment. Tumor growth is always associated with impaired antitumor immune responses, while VEGF/VEGFR are essential for tumor-induced angiogenesis and tumor growth. So VEGF/VEGFR maybe play important roles in tumor-associated immunosuppression [4].

The aim of this study intends to explore the anti-tumor effects of VEGFR-TKI combined with COX-2 inhibitor (celecoxib) in RCC therapy and the possible immunological mechanisms.

## RESULTS

### Survival of human renal cancer cells was obviously restrained

**Figure 1:**
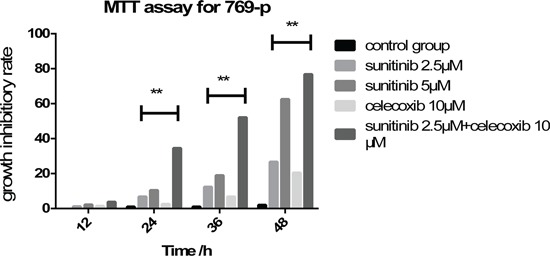
Application of sunitinib and celecoxib individually or in combination inhibits the cell viability of 769-p in a dose and time dependent manner (**P < 0.01)

MTT assay revealed that 769-p was suppressed by celecoxib or sunitinib solely or in combination in a dose and time-dependent manner. All tested 769-P exhibited similar cytotoxicity at the concentrations of sunitinib 2.5 or 5μM. Although the decrease of cell proliferation started at the concentrations of sunitinib 5μM after 24h, the inhibition was much more significant in combination of celecoxib 10μM and sunitinib 2.5μM after 48 hours incubation (Figure 1).

### Expression of GM-CSF in 769-p was substantially decreased

**Figure 2:**
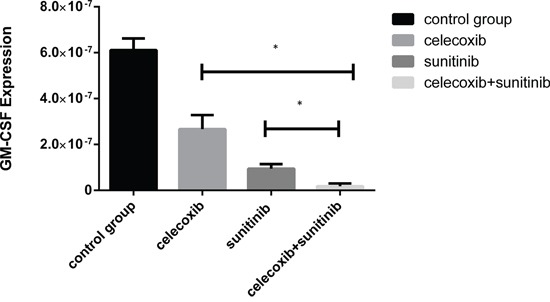
The overexpression of GM-CSF in 769-p was substantially suppressed by celecoxib and sunitinib(*P < 0.5)

Renal carcinoma cells were discovered to express a high level of endogenous granulocyte-macrophage colony-stimulating factor (GM-CSF). The expression of GM-CSF in 769-p was tested by RT-PCR and disclosed that the over expression of GM-CSF was substantially suppressed by celecoxib and sunitinib (Figure 2).

### Tumor growth in mice was strongly suppressed

One mouse in combined therapy group demonstrated the side reactions of hair loss and skin rash 15 days after treatment, while two mice in control group showed tumors decay with obvious ulcer in site of tumor growth. To which, the values of these tumor volumes were excluded. However, the blood and spleen samples were remained for analysis.

**Figure 3:**
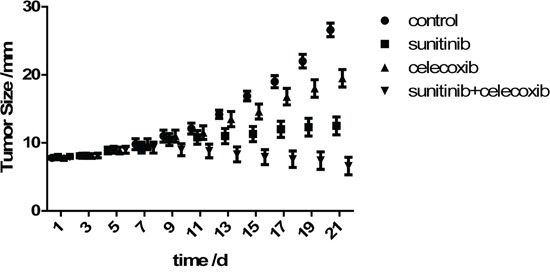
Growth curves of tumor size (long axis) in Renca model (Mean±SD)

The tumor growth in mice either treated with sunitinib alone or celecoxib added were significantly inhibited, being compared with those in control group after 1 or 2 weeks treatment (Figure 3). These results confirm that sunitinib or celecoxib monotherapy has antitumor benefits *in vivo*. However, the combined therapy possesses the greater effects on inhibition of tumor growth.

### MDSCs and Tregs in peripheral blood and spleen were significantly decreased

**Figure 4:**
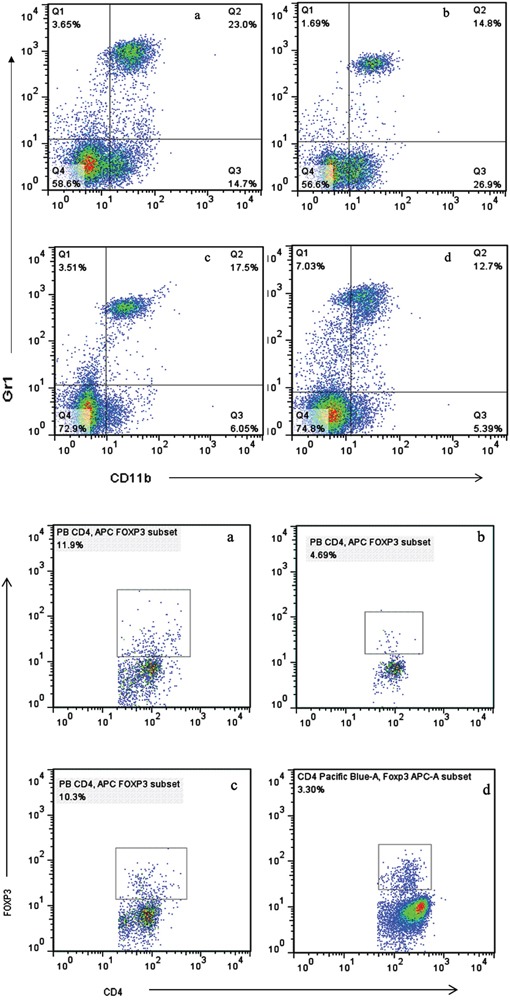
Levels of MDSCs (CD11b^+^Gr1^+^) **A.** and Tregs (CD4^+^FoxP3^+^) **B.** in peripheral blood, analyzed by multicolor fluorescence cytometry. a Control, b Sunitnib, c Celecoxib, d Celecoxib+Sunitnib.

**Figure 5:**
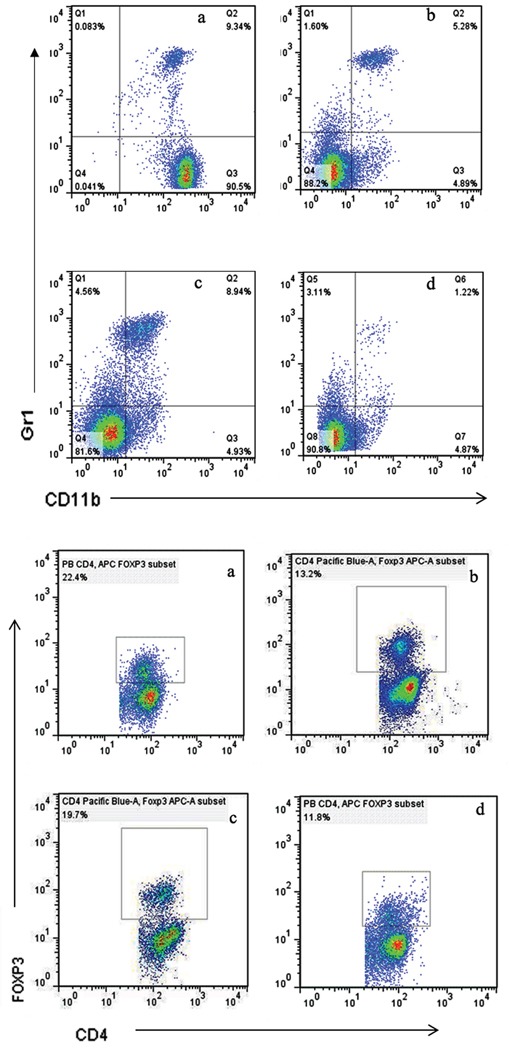
Levels of MDSCs (CD11b^+^Gr1^+^) **A.** and Tregs (CD4^+^FoxP3^+^) **B.** in spleen, analyzed by multicolor fluorescence cytometry. a Control, b Sunitnib, c Celecoxib, d Celecoxib+Sunitnib.

The populations of MDSCs and Tregs are routinely elevated in patients with progressive cancer. We evaluated the status of immunologic suppression in experimental animals by counting the MDSCs and Tregs using flow cytometry on day 21 after treatment. We found that concurrent administration of sunitinib and celecoxib can considerably reduce the MDSCs than those in control group (spleen:1.22±0.15 % vs 9.34±0.58 %, P <0.01; peripheral blood: 12.7±0.85 % vs 23.0±1.68 %, P <0.01, Figure 4), as well as decline the Tregs (spleen:11.3±1.69 % vs 22.4±2.31 %, P <0.01; peripheral blood:3.30±0.64 % vs 11.9±1.53 %, P <0.01, Figure 5). In addition, celecoxib alone showed slight decrease of Tregs and MDSCs even though without the statistic significance.

### CD4^+^ T cell level in peripheral blood and spleen were apparently elevated

**Figure 6:**
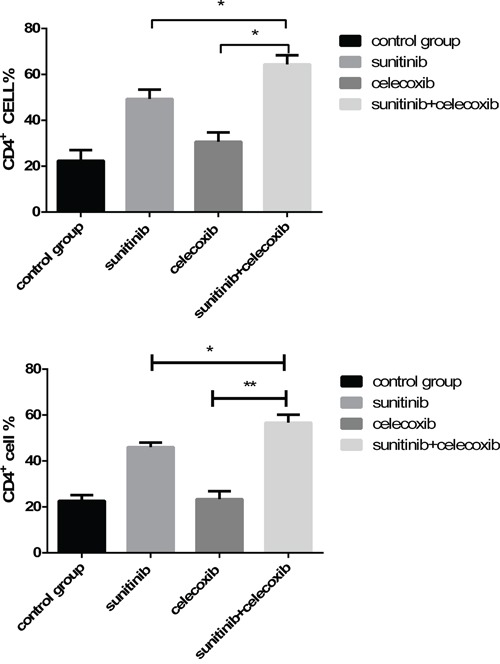
Levels of CD4^+^ T cell in peripheral blood **A.** and spleen **B.**, analyzed by multicolor fluorescence cytometry. (*p < 0.05; **p < 0.01).

Previous studies demonstrated that Tregs and MDSCs depletion could enhance the cell immunity as evidenced by the increase of CD4^+^ T cells. In our study, we found that the combination of celecoxib and sunitinib led to remarkably increase of CD4^+^ T cells in both peripheral blood and spleen than those in monotherapy group (Figure 6). It was also accompanied with a decrease of MDSCs and Tregs simultaneously. These results indicate that celecoxib may boost the antitumor activity of sunitinib via promoting T cell immunity.

### Expression of STAT3 in MDSCs were significantly reduced

**Figure 7:**
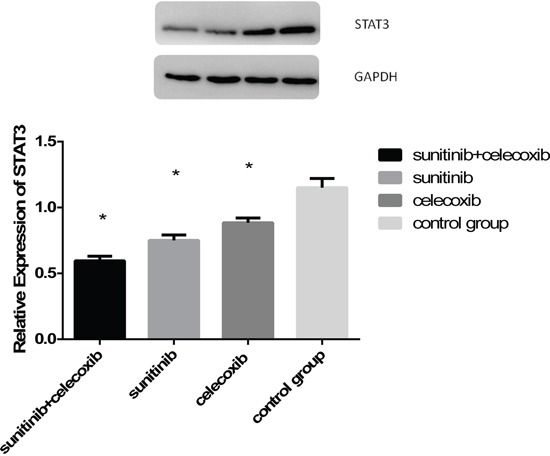
**A.** Western blot measured STAT3 expression in MDSCs from spleen as followed: combined group, sunitinib group, celecoxib group, control group; **B.** Quantitative analysis of STAT3 expression in MDSCs from spleen. (*p < 0.05).

It is well known that MDSCs is regulated via STAT3 pathways. Previous studies showed that axitinib, an alternative VEGFR inhibitor, had the potential to modulate antitumor immunity by suppressing STAT3 expression and reversing MDSCs mediated tumor-induced immunosuppression. Therefore, we try to gain the insight into the mechanisms of MDSCs reduction induced by concurrent therapy of sunitinib and celecoxib. MDSCs were isolated from spleens of experimental animals and then analyzed by western blot. The results indicated that STAT-3 were decreased in group of combined therapy (Figure 7).

## DISCUSSION

Sunitinib is one of the most promising VEGFR-TKI in mRCC treatment, which has demonstrated the effective anti-angiogenesis and antitumor activity in our primary study *in vivo*. It hinders the proliferation of RCC cells and initiated apoptosis [5]. However, patients who are refractory to the targeted therapy remain poor prognosis. Thus, it is necessary to investigate the mechanism of how it works for further developing the VEGFR TKIs therapy in mRCC.

Recent study showed that sunitinib in combination with α-GITR could induce the significant infiltration of immune cells in tumor-metastatic liver, where the cell proliferation, activation and effector of CD8^+^ T cells and/or NK cells were extremely prominent [6]. The sequence of administration of immune treatment and targeted therapy is important. In addition, mRCC patients, who discontinued the treatment of PD-1 inhibitor either in monotherapy or combined with CTLA-4 inhibitor/VEGFR-TKI, could still get the benefits from subsequent VEGFR-TKI therapy without increase of toxicity [7]. Accumulating evidence suggests that RCC is amenable to immunotherapy.

COX-2 is an important enzyme in the pathway for production of prostaglandin E2 (PGE2) and arachidonic acid. This pathway is known to play a pivotal role in mediating of inflammation, tumor growth, invasiveness and metastasis, inhibition of apoptosis and angiogenesis. Inhibition of COX-2 showed obvious antitumor and anti-angiogenic effects in some kinds of tumors, including RCC [8]. Our previous study revealed that the effects of combined therapy of IFN-α and celecoxib were prominent in mRCC patients with high COX-2 expression tumors. It suggested that COX-2 inhibition may contribute to TKI therapy.

On the other hand, tumor growth is often associated with the development and maintenance of an immunosuppressive tumor microenvironment [9]. MDSCs and Tregs are major components of immune suppressive tumor microenvironment [10]. MDSCs act to suppress antitumor immunity through a variety of mechanisms. The activation of T-cell can be suppressed by productive reactive oxygen species and arginase, which induce the production of Tregs.

The information of concurrent administration of TKI and COX-2 inhibitors in renal carcinoma is seldom. Wang et al reported that celecoxib could elevate the therapeutic effects of sunitinib, prolong the PFS of athymic mice with renal carcinoma. Meanwhile, it was found that the over expression of COX-2 was associated with the emergence of sunitinib resistance [1]. Although the efficiency of combined therapy is shown, the mechanism remains unclear. The experimental model of nude beige mice xenografts might have advantages in detection of the tumor growth, however, it could not represent the change of immune environment of human being.

In our study, the tumor size was considerably decreased in the mice treated with sunitinib and COX-2 inhibitor concurrently than those of monotherapy. The curative efficacy generally appeared in a week. Nevertheless, increasing the dosage of sunitinib to 40mg/kg in 3 weeks usually demonstrated greater therapeutic effects.

We anticipate that the molecular mechanisms of MDSCs depletion relate to sunitinib involve the blockade of STAT3 and the lessening of VEGFR expression. The activation of STAT-3 stimulated expression of proliferative genes in immature myeloid cells, leading to their development into MDSCs subsequently. VEGFR interacts with its corresponding VEGF ligand expressed on tumor cells. Blockade of these two factors may result to the diminishment of circulating MDSCs, with a lower capacity to migrate to the tumor site. The effects of sunitinib and celecoxib observed in this study can be ascribed to the significant suppression of STAT3 due to the reduction of MDSCs.

GM-CSF is known as a potent hematopoietic growth factor for granulocyte and macrophage expansion, which is able to regulate the differentiation of macrophages and granulocytes, development of dendritic cells, proliferation and activation of T cells. Our Previous study showed that GM-CSF was also a potentially independent prognostic biomarker for cancer recurrence and one of the most important factors produced by tumor cells leading to MDSCs expansion [11]. The high level of GM-CSF is required to efficiently differentiate the bone marrow cells to MDSCs. We found that the overexpression of GM-CSF in 769-p was obviously inhibited by celecoxib and sunitinib, indicating this pathway might be the site that celecoxib and sunitinib worked on *in vitro* for reducing the MDSCs and interfering the immune environment.

Taken together, our study reveals a synergistic effect of combined therapy with sunitinib and celecoxib in generating an immunological environment favorable to tumor regression. Meantime, the results provide the compelling rationale for the clinical application to the renal cell carcinoma by improving the effectiveness of immunotherapy.

## MATERIALS AND METHODS

### Cell culture

The Renca, a murine RCC cell line with a BALB/c mouse background was obtained from the American Type Culture Collection (ATCC, Manassas, VA). The human renal carcinoma cell line, 769-p, was provided by the Department of Biochemistry, Fudan University (Shanghai, China). All cells were cultured in RPMI 1640 (Gibco/Invitrogen, Grand Island, NY, USA) containing 10 % fetal bovine serum (Gibco/Invitrogen) at an incubator (37°C, 5 % CO_2_).

### Cell proliferation assessment

Cell proliferation was determined by a 3-(4, 5-dimethyl thiazol-2-yl)-2, 5-diphenyl tetrazolium bromide (MTT) assay. 1 × 10^5^ cells were seeded in each well of a 96-well plate (BD Falcon, Franklin, NJ) and allowed to adhere for 8 h. MTT solution was then added to each well at the final concentration of 0.5mg/mL and incubated for 4 hours at 37°C. After removing the culture medium, 150μL of dimethyl sulfoxide (DMSO), sunitinib and celecoxib were added respectively, and the optical density of each well was measured by spectrophotometer at 495 nm.

### Animals

BALB/c mice were purchased from Slacaboratory Animal Company (Shanghai, China) and maintained at the Department of laboratory Animal Science, Fudan University. The study project was approved by the Animal Ethics Committee of Zhongshan Hospital, Fudan University to ensure meeting the all provisions for animal experiments.

### *In vivo* tumor model

BALB/c mice age of eight weeks were anesthetized with isoflurane (0.5–1.5 volume percent) and injected with Renca cells 1 × 10^6^ (in 0.1 ml PBS) at the left flank subcutaneously. The animals were monitored for survival and allowed the tumor growth for 14 days. In general, the tumor diameter of 12mm was accepted as the starting size of pre-specified treatment because this size was comparable to a lesion in the human proportionately. Furthermore, it was an optimal size for tumor perfusion imaging. Two weeks after tumor implantation, all mice were divided into 4 groups (each group containing at least 10 mice) and treated according to the schedule as follows: sunitinib or/and celecoxib (generously supplied by Pfizer, SU-11248 AKA PF-2783926-41, Pfizer, USA) (treatment groups) and PBS (control group) were administrated orally by gavage every other day from day 0 to 28. The dosage of sunitinib 40 mg/kg/d was used based on the similarities of bioavailability and metabolism in both mouse and human in previous studies. Tumor size was measured every other day using calipers. Spleen, blood and tumor tissues were harvested at 28th day after Renca cells implantation.

### Real-time PCR

Target cells were homogenized in TRIzol reagent (Invitrogen, Carlsbad, CA) and total RNA was extracted following the manufacturer's instructions. A reverse transcription–polymerase chain reaction (RT-PCR) procedure was applied to determine the relative quantities of mRNA (One-step RT-PCR kit, Qiagen, Hilden, Germany). Twenty-eight PCR cycles were adopted for all analyses. Primers and probes were obtained from Applied Biosystems. Human or mouse glyceraldehyde-3-phosphate dehydrogenase (GAPDH) was used as an internal control. All reactions were tested in triplicate and relative expressions of RNAs compared with control samples were calculated using the ΔΔCt method.

### Flow cytometry

Peripheral blood mononuclear cells (PBMCs) were isolated from whole blood by Ficoll density gradient centrifugation and stained with anti-mouse CD4, CD25, Foxp3 and CD11b. Foxp3 staining was performed intracellularly in accordance with the manufacturer's instructions. Antibodies were purchased from BD Bioscience (BD Bioscience, San Diego, CA, USA). The number of Treg cells was expressed as the percentage of Foxp3^+^ cells within the CD3^+^CD4^+^CD25^+^ T-cell gate. The absolute number of CD4^+^ T cells was calculated from the whole blood cell count and flow cytometric analysis. Samples were analyzed with fluorescence microscope system (LEICA DMRXA, Germany). Data were analyzed using FlowJo software version 7.2.5 (TreeStar, Ashland, USA).

### Western blot analysis

Western blot was implemented as described earlier [12]. Proteins were extracted in a lysis buffer (30mmoL/L Tris, pH7.5, 150mmol/L sodium chloride, 1mmol/L phenylmethyl- sulfonylfluoride (PMSF), 1 mmol/L sodium orthovanadate, 1% Nonidet P-40, 10% glycerol, and phosphatase and protease inhibitors). The protein sample was electrophoresed by SDS–PAGE and transferred onto nitrocellulose membranes. After blocking with 5% non-fat milk, the membrane was incubated with a primary antibody followed by a horseradish peroxidase-conjugated secondary antibody. Detection was implemented with the aid of a LumiGLO chemiluminescent substrate system (KPL, Guildford, UK).

### Statistical analysis

All data were expressed as median± S.D. Nonparametric Kruskal-Wallis or Wilcoxon tests were used to analyze continuous variables. For tumor growth, all individual data were analyzed concomitantly using nonlinear mixed effect models, which allowed to share the information across subjects. Correlation between continuous variables was assessed by Spearman correlation. P-values <0.05 was considered statistically significant for overall intergroup comparisons and two-by-two comparisons. Analyses were performed with SAS software version 9.2 (SAS Institute, USA).

## Abbreviations

COX-2: cyclooxygenase-2
GM-CSF: granulocyte-macrophage colony-stimulating factor
MDSCs: myeloid-derived suppressor cells
Tregs: regulatory T cells
STAT3: signal transducer and activator of transcription 3
VEGFR: vascular endothelial growth factor receptor
TKI: tyrosine kinase inhibitor
mRCC: metastatic renal cell carcinoma
PGE2: prostaglandin E 2
PD-1: Programmed death-1
CTLA-4: cytolytic T lymphocyte associated antigen-4
ATCC: American Type Culture Collection
PBMCs: Peripheral blood mononuclear cells
